# The collaboration of surgical education fellows’ (CoSEF) guide to designing, executing, and publishing surgical education research

**DOI:** 10.1007/s44186-025-00471-6

**Published:** 2026-01-29

**Authors:** Ariana Naaseh, Sarah Lund, John M. Woodward, Joshua Roshal, Steven W. Thornton, Nicole M. Santucci, Maya L. Hunt, Sergio M. Navarro, Caitlin Silvestri

**Affiliations:** 1 Department of Surgery, Washington University in St. Louis School of Medicine, 660 South Euclid Avenue, St. Louis, MO 63110 USA; 2 Department of Surgery, Mayo Clinic, Rochester, MN USA; 3 Department of Surgery, University at Buffalo, Buffalo, NY USA; 4 Department of Surgery, Brigham and Women’s Hospital, Boston, MA USA; 5 Department of Surgery, University of Texas Medical Branch, Galveston, TX USA; 6 Department of Surgery, Duke University Health System, Durham, NC USA; 7 Department of Surgery, Indiana University School of Medicine, Indianapolis, IN USA; 8 Department of Surgery, University of Minnesota, Minneapolis, MN USA; 9 Department of Surgery, Columbia University Irving Medical Center, New York, NY USA

High-quality education research plays a critical role in improving how we train future surgeons, yet many early-career educators face challenges when launching this type of work. Limited research experience, difficulty identifying collaborators, uncertainty around methodology, and lack of protected time are just a few of the common barriers. For researchers just starting out in surgical education (from medical students to early-career faculty), the leap from idea to impactful scholarship can feel overwhelming.

This guide aims to provide a practical roadmap for key barriers the novice researcher may face when designing, executing, and disseminating meaningful surgical education research (Table 1). This perspective is not intended to be comprehensive; rather we share pragmatic insights drawn from novice to experienced researchers from a group of multi-institutional surgical residents in the Collaboration of Surgical Education Research Fellows (CoSEF).

**Table 1:** Key barriers and facilitators of conducting surgical education research

Steps in the research process	Barriers	Facilitators	Resources
Designing a study	Inexperience with particular research methods Limited exposure to research literature Difficulty aligning research questions with appropriate methods Challenges with aligning educational innovation with scholarship standards	Prior exposure to research methods shaping preferred approaches to science Experience with prior education research guiding future goals Early involvement of methodological experts Use of established frameworks	Education journals Education webinars Education conferences Mentorship Reporting guidelines (STROBE, CONSORT) Methodology primers in online journals (GSE, JSE, MedEdPORTAL)
Finding mentorship	Being new to a community Unclear interests clouding who to connect with Ambiguous goals limiting the guidance that can be provided	Prior relationships facilitating sponsorship and new connections Near peers in training or early faculty roles Thought leaders in senior positions institutionally or societally	Local interest groups Local research groups Education webinars (PEERS, ASE/SIMPL) Education conferences Structured mentorship programs (SERF, ASE committees)
Acquiring funding	Lack of experiences with grant applications Limited institutional grant infrastructure	Mentorship with a track record of funding Fundable ideas Preliminary data	Institutional grants Society grants Federal grants Grant writing workshops Institutional grant writing support resources
Collaborating with a team	Potentially limited interest locally Challenges coordinating across time zones, institutions, or busy schedules	Existing institutional research infrastructure Clear authorship agreements Defined roles and timelines	Multi-institutional collaboratives (CoSEF, SIMPL, PEGASUS, CSTAR, SERC)
Dissemination	Lack of experience with academic writing Inability to afford open access fees Lack of clarity about what different journals look to publish	Academic writing skills Funding or institutional partnerships for open access fees Familiarity with journal content preferences Conference associated journal partnerships	Grants Journals’ Guides for Authors Associate Editors

## Research mentorship and collaboration

For early-career researchers, mentorship and collaboration are foundational to identifying a clear research question and selecting an appropriate methodology. Most impactful surgical education research questions start with identifying a challenge or question in the clinical space, then iteratively refining the question with review of existing literature, support from a mentor, and identification of practical methodology. A trusted mentor and surgical education scholar is invaluable to execute novel and impactful studies. We suggest identifying a faculty member with experience in surgical education research and a history of effective mentorship with prior trainees. Near-peer mentorship (e.g., senior resident, research fellow) can be critically important when struggling to find a faculty mentor [1–3]. As CoSEF, we have successfully leveraged our broad near-peer network of trainees to unite diverse expertise across education research domains [4–8]. Faculty mentors, sponsors, and near-peers, even those without education expertise, can provide important guidance for institution-specific nuances (for example in the Institutional Review Board (IRB)) and how to overcome common roadblocks. If a mentor in your area of interest is not accessible at your institution, a thorough literature review on your research topic can help identify experts to contact.

If you are unsure of where to start in designing a study, attending education conferences and engaging with national organizations through committees can provide opportunities to network, identify mentors, and contribute to multi-institutional projects [9, 10]. Professional development programs like the Association of Surgical Education (ASE) Surgical Education Research Fellowship (SERF) provide mentorship and structured research training for faculty and trainees who struggle to find time or funding for formal advanced degrees [11, 12]. Additionally, accredited and non-accredited surgical education fellowships can provide structured educational mentorship, and training [13, 14]. However, attending conferences, participating in SERF, and completing surgical education fellowships require institutional buy-in and funding, which is limited. Online webinars like the Surgical Education Research Webinar Series and the ASE CoSEF Peer Engagement for Education Research Success Webinar Series are free opportunities to engage in learning about foundational research topics and hear from experts in the education space.

Knowing the right, and wrong, way to conduct a study is also challenging, particularly if mentorship in a specific methodology (e.g., qualitative approaches) is not readily available. If expertise in a given discipline is not available locally, we recommend collaborating across disciplines, such as within another department (e.g., internal medicine) or field (e.g., business, psychology, social science), to identify potential mentors within and outside your institution.

## Research infrastructure and study design

After identifying your mentor, the next step is to design your study and get ethical approval. In the United States, that entails determining if your educational study qualifies for IRB exemption, or requires full approval. Many educational studies qualify for IRB exemption, as they involve research (1) in an educational setting that does not impact either a trainee’s ability to learn or evaluation of instructors, (2) involving educational tests, behavioral interventions, surveys, interviews, or observation and the information is recorded in either a de-identified way or is such that identified observations will not cause harm to the research participants, or (3) supported by a department/organization that studies, evaluates, or improves an educational program. After determining if your study requires IRB approval or is exempt, the next step is to create your IRB proposal. Navigating your institutional ethical approval requires a clear understanding of guidelines and procedures, which can seem daunting without the prerequisite knowledge [15, 16]. This includes approval timelines, institutional differences in documentation requirements, and the complexities of navigating multi-institutional studies. In the United States, we recommend speaking directly to IRB personnel and reviewing the US Department of Health and Human Services Guidelines for Human Research Protections prior to submission to understand the expectations for your proposed study. Often, preparation and clear communication results in faster approvals and fewer revisions. Another great resource can be the protocols of peers and faculty at your institution who have completed similar studies with similar methodologies (e.g., survey study, qualitative interviews).

We recommend drafting a complete methodology and data analysis plan in your study proposal when requesting ethical approval. Outlining your methodology will help you identify study limitations in advance—such as inherent bias in participant selection, the inability to measure critical confounding variables, or the appropriate analyses. Often, funding is required for software or statistical support; we recommend targeting institutional grants or organizational funding. If software access is required for a specific methodological approach, we recommend exploring free or open-source software, such as Taguette (Knowledge Bank) for qualitative coding, R for quantitative analyses, or Zoom (Zoom Video Communications Inc.) for transcription services. Furthermore, some research can be more challenging to publish, including educational curricula, which requires forethought in the study design phase to ensure appropriate outcome measures are collected (e.g., assessing higher Kirkpatrick level outcomes). Reviewing a complete proposal with your mentor allows you to adjust your methodology or pivot to a different design prior to study initiation. Finally, writing a proposal upfront will save you time later; often your initial proposal frames the Introduction and Methods sections of your manuscript.

## Dissemination of scholarship

Dissemination is a critical step to maximize impact and requires strategic selection of appropriate conferences and journals [17]. Conferences offer the opportunity to disseminate your work to a local, regional, national, or sometimes international audience through presentations. Every conference is structured differently and attracts a unique audience. Work with your mentors to identify which audience your work is targeted for, and the conference that is most appropriate. It is also important to keep in mind meeting submission deadlines, types of submissions, and other conference specific requirements. An oft-unknown perk of a conference presentation is the opportunity, or requirement, to submit a manuscript to an associated journal, which typically provides a greater chance of full manuscript review or can lead to expedited publication.

Within academia, various journals exist to publish your research and are regarded differently in the hierarchy of scholarly work. Furthermore, similar to conferences, journals can have broad or targeted audiences, which should be considered. In selecting an appropriate venue, novice researchers should consult publicly available tools such as Clarivate’s Journal Citation Reports, Scimago Journal Rank, or Google Scholar Metrics to understand a journal’s impact, audience, and disciplinary reach. Journal metrics should inform, rather than dictate, journal selection, as audience is often more important than impact factor for education scholarship. Most journals provide an easily accessible ‘Aims and Scope’ page and detailed ‘Instructions for Authors’ on their websites, which offer guidance to align with the journal’s mission and submission expectations. You can also review recently published articles by the journal, which provides examples of desired rigor and content areas for submissions.

Educational scholarship lends itself to many forms of publication, including original research, perspective pieces or other formats, such as Global Surgical Education’s “Commentary,” Journal of Surgical Education’s “How I Do It,” and Medical Education’s “Really Good Stuff.” [18] When publishing a novel curricula or simulation model, consider MedEd Portal, as it focuses on publishing educational materials for teaching courses. Ultimately, surgical journals may not be the best home for your work, especially multi-disciplinary research [19]. Be open to the possibility of publishing in education, simulation, psychology, and social sciences journals. Lastly, educational scholarship takes many forms, including podcasts, webinars, workshops, and panels. A mentor can help you determine which format best aligns with your goals for dissemination of your work.

The traditional publication process is associated with logistical challenges, including costs of publication (particularly for open access work), long wait times for peer-review process, and reviewers unfamiliar with social science methodology [20]. Alongside the peer-review process, we strongly encourage you to consider pre-printing your work to reduce time to dissemination and promote greater transparency and access to research [21]. When navigating open access publications, ask if your department or mentor has funding to cover article processing charges (APCs) or if your institution’s library has partnerships with journals to waive publication fees.

## Conclusions

As interest in surgical education evolves, improved access to guidance on navigating the research process is essential to increase engagement in educational scholarship. We present a foundational approach to key barriers in the research process. Many steps in the research process (e.g. identifying a research question, clarifying study purpose, generating a hypothesis, conducting a literature review, etc.) are often iterative, rather than linear and this guide intentionally focuses on key barriers rather than addressing each discrete step in the research process.

Surgical education research demands a shift from traditional scholarship formats rooted in basic science and clinical outcomes to innovative, theory-informed methodologies and dissemination approaches that reflect its unique priorities and potential for transformative impact. We hope this practical guide empowers trainees and early-career educators to transform ideas into meaningful contributions that advance how we educate future surgeons.

## Data Availability

No new data were created or analyzed in this study. Data sharing is not applicable to this article.
